# A prospective randomized study on limits of colposcopy and histology: the skill of colposcopist and colposcopy-guided biopsy in diagnosis of cervical intraepithelial lesions

**DOI:** 10.1186/s13027-015-0042-9

**Published:** 2015-11-19

**Authors:** Giuseppe Bifulco, Nicoletta De Rosa, Giada Lavitola, Roberto Piccoli, Alessandra Bertrando, Valentina Natella, Costantino Di Carlo, Luigi Insabato, Carmine Nappi

**Affiliations:** Department of Neuroscienze e Scienze Riproduttive ed Odontostomatologiche, University of Naples “Federico II”, Naples, Italy; Department of Advanced Biomedical Science, University of Naples “Federico II”, Naples, Italy; Department of Sanità pubblica, University of Naples “Federico II”, Naples, Italy

**Keywords:** Cervical intraepithelial lesions, Multiple biopsies, Colposcopic accuracy, Colposcopic grade

## Abstract

**Background:**

The main objective of our study was to evaluate the colposcopist ability to correctly identify the worst area of a cervical lesion where biopsy should be performed; the secondary objective was to investigate the influence of the colposcopist skill in grading cervical preneoplastic lesions.

**Methods:**

296 patients referred for colposcopy were enrolled in a prospective study. All patients were randomized in two groups: in the first group, “senior group”, the colposcopy was performed by an experienced colposcopist; in the second group, “junior group”, the colposcopy was performed by a less experienced colposcopist. A detailed colposcopic description, including a grading of the lesion, was completed for each case. During the colposcopic exam patients underwent two direct biopsies; each biopsy was labeled with letter A (suspicious area with most severe grade) or B (suspicious area with less severe grade) according to the judgment of the colposcopist. An experienced pathologist reanalyzed the histological slides, after routine diagnosis.

**Results:**

The senior group identify the worst area of the cervical lesion in statistical significant higher rates than junior group. Specimen A resulted representative of the higher-grade lesion (A > B) in 73.7 % (*N* = 28) in senior group and in 48.4 % (*N* = 15) in junior group; while in 26.3 % (*N* = 10) the higher-grade lesion corresponded to specimen B (A < B) in senior group and in 51.6 % (*N* = 16) in junior group (*p* < .05).

**Conclusion:**

The ability of a colposcopist in grading cervical lesion depends on his experience.

## Background

Defining the presence, the extension and the severity of cervical intraepithelial neoplasia (CIN) is an important clinical issue in reducing cervical cancer risk and development. Colposcopy represents the second step of the diagnostic approach [1, 2]. One of the main roles of colposcopy is to guide the diagnostic biopsy. The result of the histological exam performed on the cervical biopsy is then considered as the best diagnosis in the preoperative approach to CIN.

Colposcopic accuracy varies according to the skill of the colposcopist, the age of the patients and the grade of the lesions. Mitchell and colleagues in a meta-analysis, reported that the sensitivity of colposcopy ranged from 64 to 99 % and the specificity from 30 to 93 % [3]. Indeed, colposcopic assessment more often overestimated the severity of the lesions.

Mistakes in cervical histological findings on bioptic specimens, have also been documented, [4, 5] and histological CIN diagnoses are not entirely reproducible [6, 7]. Indeed, similarly to cytological interpretation, histological assessment of cervical dysplasia is complicated by inter-observer variability [8]. The strongest source of disagreement was the threshold between normal and CIN 1. Agreement was higher for CIN 3 than for CIN 2 [9]. Moreover, the proportion of false-positive diagnoses of CIN 2 or worse varied according to cytologic and HPV test results [10].

Many studies investigated the correlation between histological diagnosis from colposcopically directed punch biopsies and definitive diagnosis after conization or hysterectomy [11, 12]. Only few authors concluded that directed cervical biopsies provide a consistent estimate of the final grading of CIN lesions [13, 14], whereas most of the studies showed just a moderate correlation. A complete agreement between biopsy and cone specimen was reported in no more than 43- 51 % of cases [11, 12, 15–19]. About 14-24 % LEEP specimens were negative for dysplasia. Giannella L. et al. showed that a severe cervical lesion (CIN2) with a minor colposcopic impression may predict a lower grade lesion on cone specimen [20].

One of the potential explanations for negative LEEP findings following a biopsy diagnosis of HSIL includes misdiagnosis of the original biopsy [21].

The choice of cervical point where to perform the biopsy is crucial to obtain a proper diagnosis. Conventionally biopsy must be performed in the worst area of cervical lesion, and should be representative of the entire lesion.

The main aim of this study was to investigate the influence of the skill of the colposcopist in correctly grading cervical preneoplastic lesion. Moreover, we investigated how good are skilled and junior colposcopists in identifying the worst area of a cervical lesion where biopsy should be performed.

## Methods

A prospective randomized study was carried out from January 2012 to October 2014, in the Unit of Cervico-Vaginal Pathology of the Department of Obstetrics and Gynecology of the University Hospital Federico II in Naples, Italy.

All women referred for colposcopic examination and undergoing cervical biopsy under colposcopic guidance were invited to participate in this study. Our Institutional Review Board approved the protocol of the study and the study was conducted according to the guidelines of the Declaration of Helsinki (1975).

After signing their informed consent, all patients with a positive cytology, were randomly assigned to two main groups (junior colposcopists and senior colposcopists) corresponding to three junior colposcopists (i.e. a post-graduate physician with one-year experience in a Unit of Cervicovaginal Pathology) and three senior colposcopists (i.e. a trained gynecologist with at least 5 years of practice in a second level Unit of Colposcopy and Cervicovaginal Pathology). A physician who was not involved in the examination used the computer-generated list to assign each patient to a colposcopist.

Colposcopy and guided cervical biopsies were performed in a single procedure.

Patients were eligible for enrolment according to both the following criteria:

- satisfactory colposcopy (squamo-columnar junction fully visible) with atypical transformation zone (aceto-white epithelium).
- aceto-white lesion extending for 2 or more quadrants (allowing the execution of a double biopsy).

A colposcopic suspect for invasive cervical cancer and pregnancy (which can alter colposcopic findings), were considered as exclusion criteria.

During colposcopic examinations, after application of 3 % acetic acid, all visible lesions were classified according to the 2011 Colposcopic Terminology of the International Federation for Cervical Pathology and Colposcopy [22].

The examiner performed, for each patient, two guided biopsies using cervical biopsy forceps with 5- to 6-mm jaws, yielding 3- to 4-mm biopsies. The two specimens were placed into two different vials of fixative.

An extensive description of the two cervical sites, where biopsies were performed, was recorded; in particular, the examiner specified:

1. the site of biopsies (dividing the cervix into 4 quadrants by 2 perpendicular lines drawn from 12 to 6 o’clock, and from 9 to 3 o’clock);
2. the grading (grade 1-minor or 2-major) and the colposcopic features (thin/dense aceto-white epithelium with fine/coarse punctuation or mosaic);
3. which biopsy was considered the most suspicious and representative of the whole cervical lesion (biopsy A) and which biopsy, performed on a less severe area of the lesion, was considered additional but not required to obtain histological diagnosis (biopsy B).

All cervical biopsies were firstly examined by the pathologist on duty at the Pathology Laboratory, who was unaware of the study. The specimens were composed of small or tiny fragments of cervical tissue. Two serial 4-μm sections of formalin-fixed, paraffin embedded samples were stained with hematoxylin and eosin. The specimens were classified according to the World Health Organization criteria as normal, CIN 1, CIN 2, CIN 3/carcinoma in situ or micro-invasive carcinoma [23].

At the end of the study all the histologic sections were reviewed by an experienced gynecologic pathologist (LI). In uncertain cases immunohistochemical stains were performed with labeling index for Ki67 to evaluate the proliferative activity, and for p16 protein expression to determine the different degrees of CIN. 4-μm serial sections from representative blocks were cut, mounted on poly-L-lysine coated glass slides and used for the immunohistochemical staining for ki67 and p16 protein. Representative sections were incubated with the primary antibodies, overnight at 4 °C. Subsequently, the slides were incubated with biotinylated secondary antibodies, peroxidase-labelled streptavidin (DAKO LSAB kit HRP, Carpinteria, CA) and chromogenic substrate diaminobenzidine (DAB, Vector Laboratories, Burlingame, U.S.A.) for the development of the peroxidase activity. Slides were counterstained with hematoxylin, dehydrated and cover-slipped with a synthetic mounting medium (Entellan, Merck, Germany).

The experienced pathologist was blinded to the referral cytology and the colposcopic examination. The histology of the most severe lesion (specimen A or B) was recoded as the final diagnosis. Although some patients underwent cervical conization or loop electrosurgical excision procedure (LEEP) as treatment for cervical neoplasia, results of these procedures were not considered in determining the final diagnosis.

### Statistical analysis

Statistical analysis was performed using SPSS software (version 20; SPSS, Inc. Chicago, IL, USA).

To compare demographic and clinical data between the two groups (senior group and junior group) Student’s t-test and Mann–Whitney test were used.

The main endpoint was to test the hypothesis that expert colposcopists may perform with a higher degree of accuracy the guided cervical biopsy. If this were true, expert colposcopists would identify the worse biopsy in a higher percentage of cases in comparison to junior colposcopists.

Differences in proportions were tested with χ^2^ test (sites of biopsies, histological diagnosis rates, gradation of colposcopist judgment attributed to biopsy sites) and with Wilcoxon’s signed rank test (routine versus revision histological analysis). Statistical analysis for colposcopist evaluation attributed to biopsy sites was performed considering only patients with definitive diagnosis of CIN. A colposcopist evaluation reporting A = B was considered not informative and excluded from the statistical analysis. The level of significance for these tests was set at *p* < 0.05.

The significance of the association between colposcopic grading and biopsy histology was determined using χ^2^ test, while the strength of the association was assessed using κ statistics. Prior to calculating the κ values the histological diagnosis were dichotomized into two classifications: Negative/Cervicites/Metaplasia/koilocytosis /Condylomatosis/CIN 1 and CIN 2/CIN 3. Standard definitions were used to interpret the κ statistics [24].

## Results

**Fig. 1:**
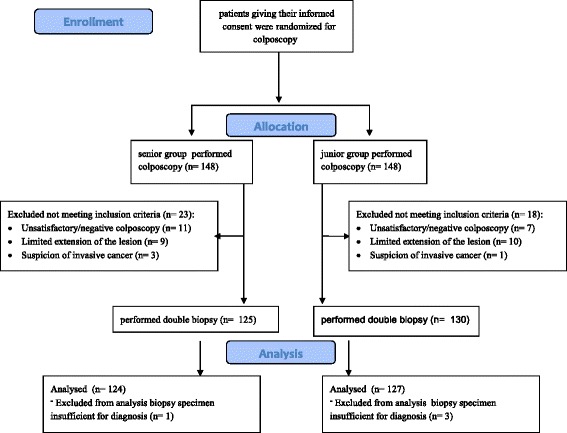
Patients enrolment and randomization

A total of 296 gave their consent to participate in this study. At the time of colposcopy, 41 patients were excluded as they did not satisfy the inclusion criteria: 18 women had unsatisfactory or negative colposcopy, 19 women had limited extension of lesion involving 0–1 quadrant of the cervix and 4 women had a suspected invasive cervical cancer. Among the 255 enrolled patients in 4 cases one or both biopsy specimens were insufficient for a diagnosis. Therefore, 251 cases met all criteria for analysis according to the study protocol (Fig. 1).

The mean age of patients was 32.4 years (range 19–52; SD ± 8.5).

One hundred twenty seven cases were randomized into senior group (50.6 %), while 124 cases (49.4 %) into junior group. Demographic characteristics of the patients, indications for colposcopy, colposcopic grade and final histological diagnosis are shown in Table 1. No significant differences for age, parity, educational level, colposcopic indications, colposcopic grade and final histological diagnosis were found between the two groups (Table 1).

**Table 1:** Patient demographic characteristics, colposcopic indication and findings, histological diagnosis for two groups

		junior group^a^ (*N* = 124)	senior group^a^ (*N* = 127)	*p Value*
Age (years ± S.D.)		32.2 ± 8.1	32.6 ± 8.9	.23
Parity (N ± S.D.)		0.62 ± 0.92	0.57 ± 0.85	.69
Educational level				.82
	Elementary education	3 (2.4)	2 (1.6)	
	Lower secondary education	50 (40.3)	51 (40.2)	
	Upper secondary education	58 (46.8)	60 (47.2)	
	Postsecondary education	13 (10.5)	14 (11.0)	
Colposcopic Indication^b^				.43
	ASC-US/ASC-H	35 (27.6)	32 (25.8)	
	AGC-NOS	4 (3.1)	5 (4.0)	
	L-SIL	57 (44.9)	66 (53.2)	
	H-SIL	31 (24.4)	21 (16.9)	
Colposcopic Grade^c^				.10
	TAG1	104 (83.9)	96 (75.6)	
	TAG2	20 (16.1)	31 (24.4)	
Final Histological Diagnosis				.91
	Negative/ Cervicites / Metaplasia	56 (45.2)	61 (48)	
	CIN1 / Koilocytosis / Condylomatosis	39 (31.5)	33 (26)	
	CIN 2/3	29 (23.4)	33 (26)	

^a^In senior group colposcopic examination and biopsies were performed by experienced colposcopists; in junior group post-graduate doctors with one-year experience in Unit of Cervicovaginal Pathology performed the diagnostic procedures

^b^Indications for colposcopy: Atypical squamous cells of undetermined significance (ASC-US); Atypical squamous cells – cannot exclude HSIL (ASC-H); Atypical Glandular Cells not otherwise specified (AGC-NOS); Low grade squamous intraepithelial lesion (LSIL); High grade squamous intraepithelial lesion (HSIL)

^c^TAG1: Atypical Transformation of Grade 1, TAG2: Atypical Transformation Grade 2

Data regarding histological diagnosis performed by the routine practice pathologist and by the experienced gynaecologic pathologist for specimen A and B are shown in Table 2. A significant statistical difference was found between routine and revised histological analysis only for specimen B (*p* = .03) (Table 2).

**Table 2:** Histological diagnosis of specimen A and B resulted from the routine analysis and from the revision analysis performed by an experienced gynecologic pathologist

Hystological Diagnosis	Specimen A N (%)			Specimen B N (%)		
	Routine Analysis	Revision Analysis^a^	*p Value*	Routine Analysis	Revision Analysis^a^	*p Value*
Negative	18 (7.2)	40 (15.9)	.34	35 (13.9)	51 (20.3)	.03
Cervicites / Metaplasia	116 (46.2)	96 (38.2)		95 (37.8)	102 (40.6)	
CIN1 / Koilocytosis / Condylomatosis	71 (28.3)	65 (25.9)		73 (29.1)	52 (20.7)	
CIN2	20 (8.0)	14 (5.6)		24 (9.6)	10 (4.0)	
CIN3	26 (10.4)	36 (14.3)		24 (9.6)	36 (14.3)	

^a^Revision analysis: analysis performed by experienced pathologist. In uncertain cases immunohistochemical stains were used, particularly, antibody against ki67 to evaluate the proliferative activity and p16 protein expression to determine the different degrees of CIN

Considering only patients with definitive histological diagnosis of CIN (diagnosis performed by the expert pathologist) the senior group identify the worst area of the cervical lesion in statistical significant higher rates than junior group. Specimen A resulted representative of the higher-grade lesion (A > B) in 73.7 % (*N* = 28) in senior group and in 48.4 % (*N* = 15) in junior group; while in 26.3 % (*N* = 10) the higher-grade lesion corresponded to specimen B (A < B) in senior group and in 51.6 % (*N* = 16) in junior group (Table 3, *p* < .05). The difference was significant both in routine than in revised histological analysis (Table 3, *p* < .05).

**Table 3:** Gradation of colposcopist judgment attributed to biopsy sites corresponding to specimen A and B before and after revision analysis in senior and junior group

Examiner group^a^	Colposcopist evaluation^b^	senior N (%)	junior N (%)	*p value*
Routine histological Analysis	A < B	10 (26.3)	16 (51.6)	.03
	A > B	28 (73.7)	15 (48.4)	
Revised histological Analysis^c^	A < B	12 (27.3)	19 (50.0)	.03
	A > B	32 (72.7)	19 (50.0)	

Statistical analysis was performed considering only patients with definitive diagnosis of CIN. A colposcopist evaluation reporting A = B was considered not informative and excluded from the statistical analysis

^a^In senior group colposcopic examination and biopsies were performed by experienced colposcopists; in junior group post-graduate doctors with one-year experience in Unit of Cervicovaginal Pathology performed the diagnostic procedures

^b^According to the judgment of the colposcopist biopsy A was considered the most suspicious and representative of the whole cervical lesion and biopsy B was considered additional but not required to obtain histological diagnosis

^c^Revision analysis: analysis performed by experienced pathologist. In uncertain cases immunohistochemical stains were used, particularly, antibody against ki67 to evaluate the proliferative activity and p16 protein expression to determine the different degrees of CIN

A significant difference was also found in rate of colposcopist evaluations between groups when stratified by colposcopic findings of grade 1 and 2 (Table 4). Indeed, in presence of grade 1 lesions, junior colposcopist identified in A a less severe lesion than in B in a significant higher rate of cases than in senior group (A < B: 70.0 % vs. 36 %, *p* = .01). The difference was not significant in grade 2 lesions (Table 4).

**Table 4:** Gradation of colposcopist judgment attributed to biopsy sites corresponding to specimen A and B stratified for colposcopic grading of the lesion in senior and junior group

Colposcopic Grading^c^	Colposcopist evluation^b^	Examiner Group^a^		*p value*
		senior group	junior group	
TAG1	A < B	9 (36.0)	21 (70.0)	.01
	A > B	16 (64.0)	9 (30.0)	
TAG2	A < B	5 (20.0)	0 (0.0)	.09
	A > B	20 (80.0)	12 (100.0)	

Statistical analysis was performed considering only patients with definitive diagnosis of CIN. A colposcopist evaluation reporting A = B was considered not informative and excluded from the statistical analysis.

^a^In senior group colposcopic examination and biopsies were performed by experienced colposcopists; in junior group post-graduate doctors with one-year experience in Unit of Cervicovaginal Pathology performed the diagnostic procedures.

^b^According to the judgment of the colposcopist biopsy A was considered the most suspicious and representative of the whole cervical lesion and biopsy B was considered additional but not required to obtain histological diagnosis.

^c^TAG1: Atypical Transformation of Grade 1, TAG2: Atypical Transformation Grade 2.

The association between histological diagnosis and colposcopic grade was highly significant (p < .001) and the strength of the correlation, as assessed by the κ statistics, was fair for each specimen (A or B) and histological analysis (κ = 0.32; CI 95 %: .16-.47; and κ = 0.30; CI 95 %: .17-.44, for routine and revised analysis respectively) (Table 5). The highest κ value was observed in senior group (κ = .42; CI, 95 %: .25-.62) (Table 5). In junior group, the association between histological diagnosis and colposcopic grade was shown significant (*p* <0.05) but the strength of this association was found slight (κ = .20; CI 95 %: −.01-.40 ) (Table 5).

**Table 5:** Association and strength of correlation between histological diagnosis and colposcopic grading in senior and junior group

		Histological Diagnosis^b^		*p value* ^*c*^ K Value; 95 % C.I.
Specimen	Colposcopic Grading^a^	Negative/CIN1 N (%)	CIN2/3 N (%)	
Routine analysis after single biopsy (A)				
total group	TAG1	176 (85.9)	24 (52.2)	*<.001* 0.32; .16-.47
	TAG2	29 (14.1)	22 (47.8)	
senior group	TAG1	87 (84.5)	9 (37.5)	*<.001* 0.42; .25-.62
	TAG2	16 (15.5)	15 (62.5)	
junior group	TAG1	89 (87.3)	15 (68.2)	*<.05* 0.20; −.01-.40
	TAG2	13 (12.7)	7 (31.8)	
Revision analysis after two biopsies (A and B)				
total group	TAG1	164 (86.8)	36 (58.1)	*<.001* 0.30; .17-.44
	TAG2	25 (13.2)	26 (41.9)	
senior group	TAG1	80 (85.1)	16 (48.5)	*<.001* 0.37; .15-.54
	TAG2	14 (14.9)	17 (51.5)	
junior group	TAG1	84 (88.4)	20 (69.0)	*<.05* 0.22; .03-.42
	TAG2	11 (11.6)	9 (31.0)	

^a^The histology of the most severe lesion obtained with specimen A or B was recoded as the final histological diagnosis

^b^TAG1: Atypical Transformation of Grade 1, TAG2: Atypical Transformation Grade 2

^c^The significance of the association between colposcopic grading and histological diagnosis was determined within group using χ^2^ test, the strength of the association was assessed using κ statistics. To perform this analysis the histological diagnosis were dichotomized into two classifications: Negative/Cervicites/Metaplasia/koilocytosis/Condylomatosis/CIN 1 and CIN 2/CIN 3

## Discussion and conclusion

This study prospectively investigates the ability of colposcopist in grading and performing diagnosis of a CIN lesion in uterine cervix.

Our data show a high significant correlation between colposcopic grading and histologic grading in single and in double biopsy both in routinely and in revision analysis as well.

This high correlation was lost when colposcopy was performed by less experienced examiner (junior Group). On the other hand, in senior group exact agreement was found in 85.1 % of grade 1 lesions and in 51.5 % of grade 2 lesions. Accordingly, the strength of this correlation, as assessed by κ statistics, is fair (κ = .42) in senior Group and slight in junior group (κ = .20). Baum ME el al. [25] and Benedet JL. et al. [26] have shown similar data in k statistic according to examiners experience. Overall the highest difficulty both in senior than junior group was the identification of grade 2 lesions.

In Benedet’s report, the association between Pap smear cytology and colposcopic impression has been found higher significant than association between punch biopsy histology and colposcopy, indeed the strength of this correlation was moderate (κ = .56) [26]. However, also the correlation between cervical punch biopsy and LEEP biopsy was moderate (κ = .44) or even fair (κ = .31) [25–28].

The value of multiple or random cervical biopsies at the time of colposcopy for evaluation of an abnormal cytology has been discussed in the last decades. The number of specimens seems to influence the sensitivity of the diagnosis on cervical biopsies. The proportion of women with CIN 2 or worse increased when multiple random cervical biopsies in quadrants without lesions were performed [27]. Zuchna C. et al. [29] showed that two biopsies achieved a highly significant improvement in agreement between punch biopsy and cone specimen in comparison to one biopsy. On the contrary, in our study, second biopsy did not increase the strength of correlation between colposcopic and histological grade neither in senior nor in junior group.

Colposcopic findings judged more representative of CIN lesion are presence of mosaics and punctuation both in colposcopic grade 1 and 2, while thin or dense aceto-white epitelium without vascular pattern are considered by colposcopist less representative of lesion grade. The ability of colposcopist in differentiating and grading two point of the same cervical lesion is limited.

In presence of a CIN lesion, the rate of colposcopic evaluations in biopsy sites (A and B) correct (A > B), was 62 %, on the contrary in 38 % of cases colposcopists do not identify the worst area of the cervical lesion (A < B).

Massad et al. [30] previously reported that colposcopic impression, colposcopic features as color, margin, vascularity and modification of Reid index do not discriminate between acetowhite lesion that arbor CIN2+ and those that not. However, the failure to detect CIN2+ lesion at colposcopy may reflects a measurable physical characteristic of the dysplastic epithelium. Yang B. et al. demonstrate that false negative colposcopic impression is sometimes secondary to the relative thinness of some CIN2-3 lesion [31].

The colposcopist experience influences significantly colposcopic accuracy in grading cervical lesion and in identify its worst area; indeed, junior colposcopist showed more difficulties in identify the worst area of the lesion, in about half of cases they fail in the classification of the lesion.

Colposcopic grade influence colposcopist judgment in less expert colposcopists; higher colposcopic lesion grade (grade 2) is associated with higher rate of correct evaluation in junior group (A > B). On the contrary junior colposcopist fail, significantly more than senior colposcopist, in identification of worst area was to perform biopsy in grade 1 lesion. Probably this is due to the larger extension of high-grade lesion and to the coexistence of low-grade area in high-grade lesion.

Overall, 42.2 % of histological reports have been modified after revision by experienced gynecologic pathologist: 20.8 % cases resulted in a reduction and 33 % showed an increase of lesion’s grade, while in 46.2 % the adjustment did not influenced the grade of the lesion. This high rate of changed reports could be attributed to the use of immunochemistry for Ki-67 and p16INK4a antibodies that have been demonstrated in many studies valuable adjunctive aids in diagnosis of difficult cervical biopsy [32–34].

A significant statistical difference was found between routine analysis and revision analysis in specimen B. These data show that the second biopsy, performed in cervical area judged by colposcopist less representative of lesion grade and characterized at colposcopy by the absence of vascular pattern and by less marked signs of lesion makes more difficult in performing histological analysis.

In conclusion, our data suggest that a long time experience in a Colposcopic Unit is fundamental to ensure high accuracy of colposcopy, and that a one year colposcopy training program is not enough to achieve these skills. Also expert colposcopist when performs a guided biopsy may not identify the worst area of a CIN lesion, so neither colposcopic impression nor histology can be used alone to guide management.

The lack of a definitive diagnosis on excisional sample (LEEP or cold knife conizzation) cannot ensure the conclusion that perform a second biopsy have a minimal impact on colposcopy accuracy, further study are necessary to achieve this goal.
